# MSPCD: predicting circRNA-disease associations via integrating multi-source data and hierarchical neural network

**DOI:** 10.1186/s12859-022-04976-5

**Published:** 2022-10-14

**Authors:** Lei Deng, Dayun Liu, Yizhan Li, Runqi Wang, Junyi Liu, Jiaxuan Zhang, Hui Liu

**Affiliations:** 1 School of Computer Science and Engineering, Central South University, Hunan, 410083 China; 2 Viterbi School of Engineering, University of Southern California, Los Angeles, 90089 USA; 3 Department of Cognitive Science, University of California San Diego, La Jolla, 92093 USA; 4 School of Computer Science and Technology, Nanjing Tech University, Nanjing, 211816 China

**Keywords:** Circrna-disease associations, Multi-source data, Neural network, High-order features

## Abstract

**Background:**

Increasing evidence shows that circRNA plays an essential regulatory role in diseases through interactions with disease-related miRNAs. Identifying circRNA-disease associations is of great significance to precise diagnosis and treatment of diseases. However, the traditional biological experiment is usually time-consuming and expensive. Hence, it is necessary to develop a computational framework to infer unknown associations between circRNA and disease.

**Results:**

In this work, we propose an efficient framework called MSPCD to infer unknown circRNA-disease associations. To obtain circRNA similarity and disease similarity accurately, MSPCD first integrates more biological information such as circRNA-miRNA associations, circRNA-gene ontology associations, then extracts circRNA and disease high-order features by the neural network. Finally, MSPCD employs DNN to predict unknown circRNA-disease associations.

**Conclusions:**

Experiment results show that MSPCD achieves a significantly more accurate performance compared with previous state-of-the-art methods on the circFunBase dataset. The case study also demonstrates that MSPCD is a promising tool that can effectively infer unknown circRNA-disease associations.

## Background

Circular RNA is a special class of single-stranded, non-coding RNA, which forms a closed covalent ring structure by connecting the 3′ and 5′ ends through exon or intron circularization. In the 1970s, circRNA was discovered for the first time in Viroids and Sendai virus particles of infected plants by electron microscopy and other technologies [1]. Later, circRNA was also found in both Animal cells [2] and fungal cells [3]. Due to the limitations of biotechnology and the particularity of structure, circRNA was considered an artifact or miss-splicing product. Therefore, in the following decades, it was not taken seriously by scientists. The past decades have seen a growth in high-throughput sequencing and other related technologies. Biologists discovered that circRNA is widely present in archaea and may have biological functions [4]. Simultaneously, Salzman et al. [5] also proved that circRNA is a general feature in the gene expression program of human cells and may play an essential role in various biological functions. Then Nature published two articles [6, 7] about the biological function of circRNA, which unveiled the mystery of circRNA for the first time. Since then, circRNA has raised the interest of many biologists. PubMed, a well-known biomedical database, has collected more than 7000 articles about circRNA from 2012 to December 2020, and it is still on the rise. Many of the articles are focused on the research of associations between circRNA and diseases due to its resistance to exonuclease-mediated degradation and higher stability than most linear RNA in cells.

Existing experimental results reveal that circRNA plays a crucial role in diseases by interacting with disease-related miRNAs and has excellent potential to become a new clinical diagnostic marker. Hense et al. [8] have confirmed that CDR1as and miR-7 are co-expressed in the mouse brain and affect midbrain development. The expression level of $hsa\_circRNA\_100855$ is higher in patients with cervical lymph node metastasis or late clinical treatment at stage T3 [9]. For the treatment of depression, $hsa\_circRNA\_103636$ is a potential new biomarker [10]. Liu et al. [11] used circRNA chips to screen the differential expression of circRNA and co-expression analysis of ceRNA between arthritis patients and normal people, concluding that circRNA-CER may be a potential target for arthritis treatment. In addition, circRNA is also closely related to atherosclerosis [12], diabetes [13], Ruan virus disease [14], viral hepatitis [15], and neurological diseases [16]. There is a strong correlation between circRNA and diseases, so recognizing their associations is essential to disease treatment and diagnosis. However, these experimental methods are expensive, difficult, and slow-progressing. An effective computational method is necessary for identifying circRNA-disease associations.

In recent years, many computational methods were proposed and mostly divided into two types: methods based on network and methods based on machine learning. As for network-based methods, Lei et al. [17] proposed a new computational path weighted method for predicting circRNA-disease associations on the circR2Disease dataset. Fan et al. [18] presented a heterogeneous network-based model, named KATZHCDA, by integrating disease similarity matrix, circRNA expression profiles, and known circRNA-disease associations and using the KATZ model to measure circRNA-disease associations. Deng et al. [19] introduced the KATZCPDA for identifying circRNA-disease associations with multiple heterogeneous networks constructed by the integrations among circRNAs, proteins, and diseases. Zou et al. [20] constructed multiple similarity networks and association networks and used the double matrix factorization method to infer circRNA-disease associations. Lei et al. [21] used the random walk with restart algorithm to weight the features and then used the k-nearest neighbor to predict unknown circRNAs and disease associations.

As for methods based on machine learning, Zheng et al. [22] provided iCDA-CGR based on non-linear information and quantify location to predict the circRNA-disease associations. The method first uses Chaos Game Representation to quantify the non-linear sequence relationship of circRNA with biological sequence position information. Fan et al. [23] presented a novel approach MSFCNN using CNN. The similarity kernels of circRNA or disease are integrated with the similarity kernel fusion method. Then, they constructed the feature matrix using interaction features and multiple similarity kernels among diseases, miRNAs and circRNAs. Finally, the model predicts potential circRNA-disease association by trained CNN with the features matrix input. Wang et al. [24] proposed a new model implemented by extreme learning machine and CNN. The CNN model is constructed for effective hidden-feature extraction, and the ELM classifier is designed to identify potential circRNA-disease association on the circR2Disease dataset. Xiao et al. [25] presented a method by graph-based multi-label learning to predict potential associations. The model contains the graph regularization and mixed-norm constraint terms to make a better prediction. Wei et al. [26] provided iCircDA-MF using matrix factorization to predict the circRNA-disease associations. Zhao et al. [27] developed a novel method IBNPKATZ with KATZ measure and the bipartite network projection. Lei et al. [28] designed a model using gradient boosting decision tree to predict circRNA-disease associations on the circR2Disease dataset. Wang et al. [29] developed a new model based on Fast learning with graph convolutional networks (FastGCN). Ding et al. [30] presented an approach by logistic regression model and the random walk. Chen et al. [?] first constructed multiple association networks, then integrated multiple similarities to generate circRNA and disease features, and then used graph attention network to predict circRNA-disease associations. The association between disease and circRNA is a complex process involving plentiful biological information. Due to the lack of full use of relevant biological information, the performance of the above models has much room to improve.

In this work, we develop a method called MSPCD, which calculates circRNA similarity and disease similarity more accurately by integrating more biological information such as circRNA-disease associations, circRNA-gene ontology (GO) associations. MSPCD also utilizes the neural network to extract circRNA and disease high-order features and adopts the DNN to infer unknown circRNA-disease associations. To verify the performance of MSPCD, five-fold cross-validation is performed on the circFunBase dataset. The AUC value of MSPCD is 0.9904 on the circFunBase dataset. Moreover, we compare MSPCD with several state-of-the-art computational frameworks on the circFunBase dataset and perform a case study. The experimental results demonstrate that MSPCD is efficient in inferring unknown circRNA-disease associations.

## Results and discussion

### The performance of MSPCD based on five-fold cross-validation

**Fig. 1:**
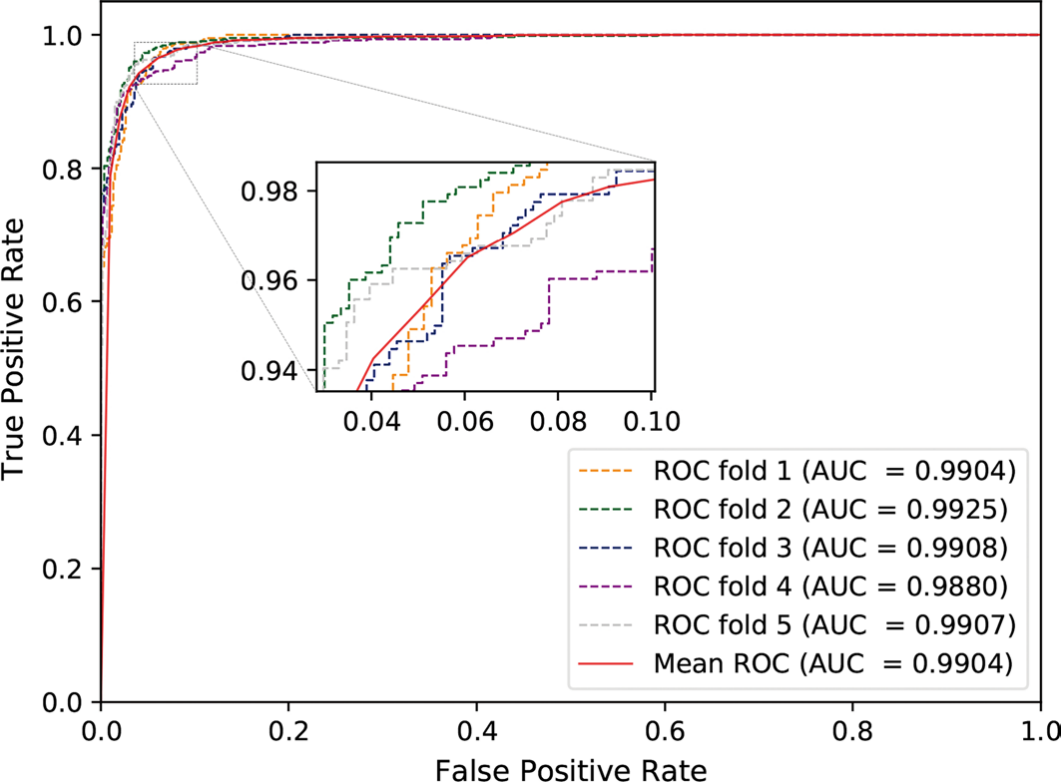
ROC curves performed by MSPCD on circFunBase dataset

To evaluate MSPCD’s predictive ability in circRNA-disease associations, we implement five-fold cross-validation experiments on the circFunBase dataset. The experimental results are summarized in Table 1. In addition, we also plot the *ROC* curve, as shown in Fig. 1. From the table, we can see that the five experiments’ *AUC* values of the MSPCD model reach 0.9903, 0.9924, 0.9908, 0.9879, 0.9907, respectively, and the average value is 0.9904. In terms of accuracy index, the five experiments’ accuracy values are 0.9505, 0.9631, 0.9296, 0.9371, and 0.9463, respectively, with an average value of 0.9453. The $F1\_score$ reflects a harmonic average of the accuracy and recall rate of the model. The $F1\_score$ values of the five experiments are 0.9501, 0.9652, 0.9314, 0.9387, 0.9473, respectively, and the average value is 0.9463. Besides, the average *precision* and *recall* are 0.9246 and 0.9702, respectively. The above experimental results demonstrate that MSPCD has good performance in inferring unknown circRNA-disease associations.

**Table 1:** Five-fold cross-validation results on circFunBase dataset

Validation set	AUC	Accuracy	Precision	Recall	F1_score
1	0.9903	0.9505	0.9461	0.9541	0.9501
2	0.9924	0.9631	0.9546	0.9760	0.9652
3	0.9908	0.9296	0.8811	0.9878	0.9314
4	0.9879	0.9371	0.9259	0.9519	0.9387
5	0.9907	0.9463	0.9157	0.9812	0.9473
Average	0.9904	0.9453	0.9246	0.9702	0.9463

### Comparison with existing state-of-the-art methods

In this section, we compare the model with several state-of-the-art methods (DMFCDA [31], KATZCPDA [19], AE_RF [32], GBDTCDA [28], IMS-CDA [33], AE_DNN [34]). DMFCDA regards circRNA-disease associations prediction as a kind of recommendation problem. Firstly, DMFCDA extracts circRNA and disease latent features from the original circRNA-disease association matrix, respectively, then cascades circRNA and disease latent features to represent circRNA and disease pair. Finally, DMFCDA utilizes a DNN to realize the prediction of circRNA and disease associations. KATZCPDA first integrates multiple heterogeneous networks including circRNA, protein, and disease, and then uses the KATZ method to predict the relationship between circRNA and disease. AE_RF firstly obtains circRNA similarity and disease similarity by integrating circRNA functional similarity, circRNA Gaussian interaction profile kernel similarity, disease semantic similarity and disease Gaussian interaction profile kernel similarity. Then, AE_RF uses autoencoder for feature selection, and employs Random Forest to give the final circRNA-diseases association predictions. GBDTCDA uses circRNA-related expression profiles, circRNA sequences, and gene ontology (GO) terms data to construct a circRNA similarity network, and then uses the GBDT algorithm to identify circRNA-disease associations. IMS-CDA first combines the semantic similarity of diseases, Jaccard similarity and Gaussian interaction profile kernel similarity of disease and circRNA. Then IMS-CDA uses stacked autoencoder to extract latent features. Finally, the random forest classifier is used to predict the association between circRNA and disease. AE_DNN resembles the AE_RF and have several vital improvements. AE_DNN replaces circRNA functional similarity with circRNA sequence similarity when integrating circRNA similarity, and replaces the RF classifier with the DNN classifier.

**Table 2:** The comparison of different methods based on five-fold cross-validation

Model	AUC	Accuracy	Precision	Recall	F1_score
MSPCD	0.9904	0.9453	0.9246	0.9702	0.9463
DMFCDA	0.9492	0.8954	0.8816	0.9149	0.8978
KATZCPDA	0.9208	0.9103	0.9204	0.8837	0.9016
AE_RF	0.9079	0.9079	0.9689	0.8426	0.9006
GBDTCDA	0.9064	0.8899	0.9004	0.8603	0.8798
IMS-CDA	0.8773	0.8403	0.8771	0.8156	0.8452
AE_DNN	0.7816	0.7055	0.7707	0.6024	0.6649

**Fig. 2:**
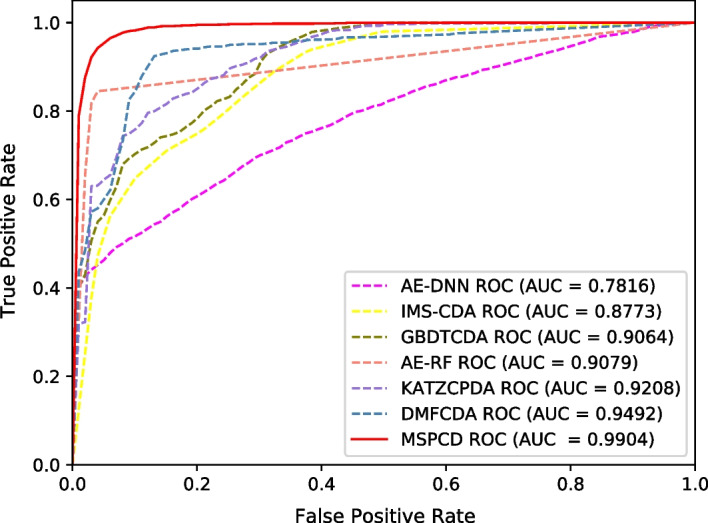
ROC curves performed by different methods on circFunBase

We perform five-fold cross-validation experiments. Table 2 shows experimental results for the seven methods. Furthermore, we also plot ROC curves, as shown in Fig. 2. From the table, we can see that MSPCD *AUC* value achieves the best result, which is much higher than that of the second-best method by 4.12%. And compared with other evaluation indicators, it has also achieved the best results on *accuracy*, *recall* and $F1\_score$. Our model can achieve such good results because it not only integrates additional biological information to obtain circRNA similarity and disease similarity, but also utilizes hierarchical neural networks to predict circRNA-disease associations.

**Fig. 3:**
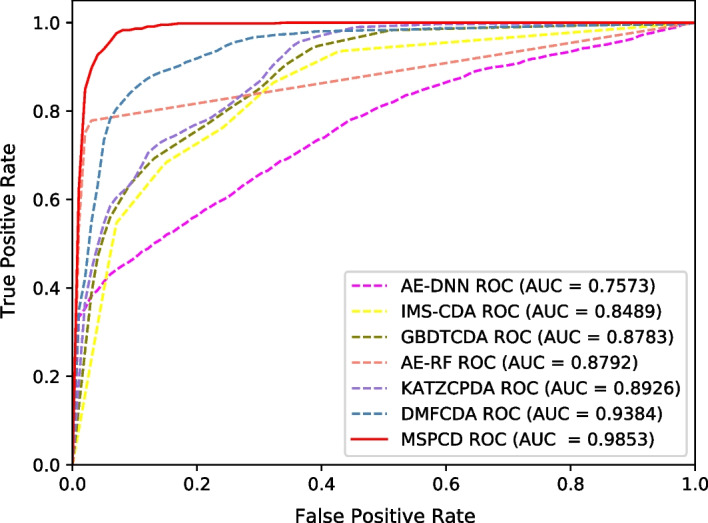
ROC curves performed by different methods on independent testing dataset

In order to further evaluate the performance of MPSCD, we conduct experimental comparisons on an independent testing dataset. We randomly select 20% of the samples from the circFunBase dataset as the independent testing dataset, which is, 2984*20% $\approx$ 596 samples. The remaining $2984-596$ = 2388 samples are divided into five parts of roughly the same size, which are used in the training dataset and the validation dataset to cross-validate the model. After this division, we ensure that the independent testing dataset does not overlap with other datasets. We draw the ROC curve of these methods on the independent testing dataset. From Fig. 3, we can see that the AUC value of MSPCD is 0.9853 on independent testing dataset.

### Comparison of different datasets

**Fig. 4:**
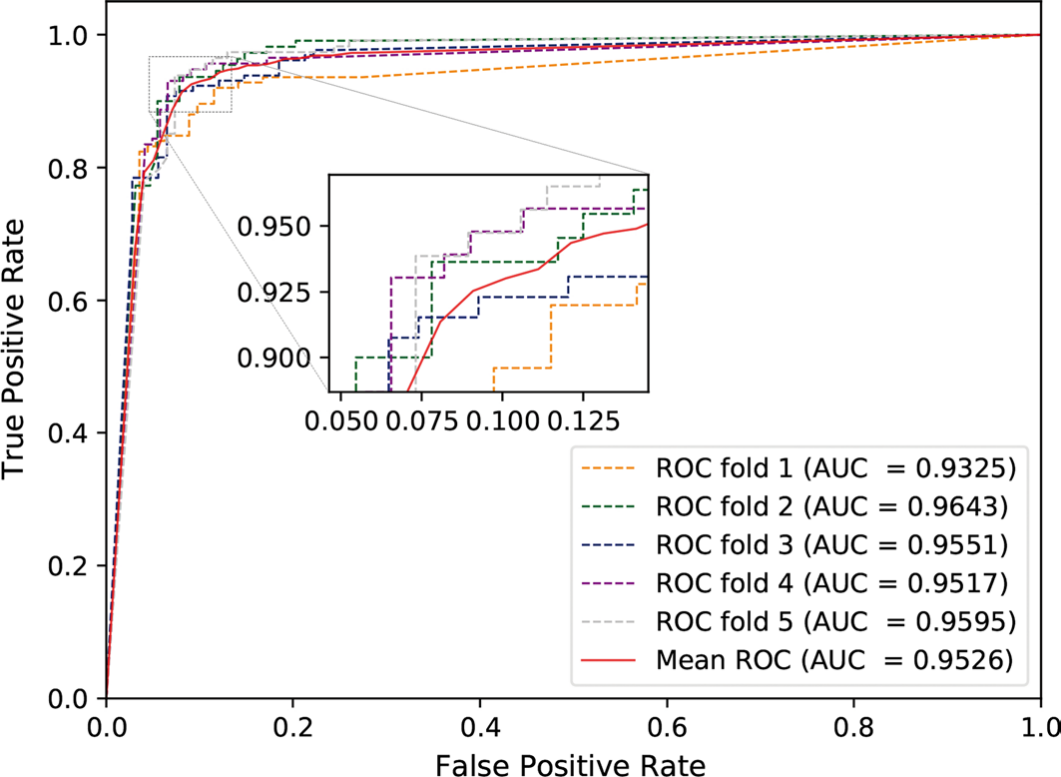
ROC curves performed by MSPCD on circR2Disease dataset

To further verify the performance of MSPCD, we perform a five-fold cross-validation experiment on the circR2Disease dataset. The experimental results are illustrated in Table 3, and the *ROC* curve is shown in Fig. 4. From the table, we can see that the average *AUC* value, *accuracy*, *precision*, *recall*, $F1\_score$ of MSPCD on circR2Disease are 0.9526, 0.9157, 0.9119, 0.9219, and 0.9163, respectively.

**Table 3:** Five-fold cross-validation results on circR2Disease dataset

Validation set	AUC	Accuracy	Precision	Recall	F1_score
1	0.9325	0.8907	0.9090	0.8800	0.8943
2	0.9643	0.9285	0.9115	0.9363	0.9237
3	0.9551	0.9201	0.9370	0.9153	0.9260
4	0.9517	0.9240	0.9292	0.9130	0.9210
5	0.9595	0.9156	0.8730	0.9649	0.9166
Average	0.9526	0.9157	0.9119	0.9219	0.9163

**Fig. 5:**
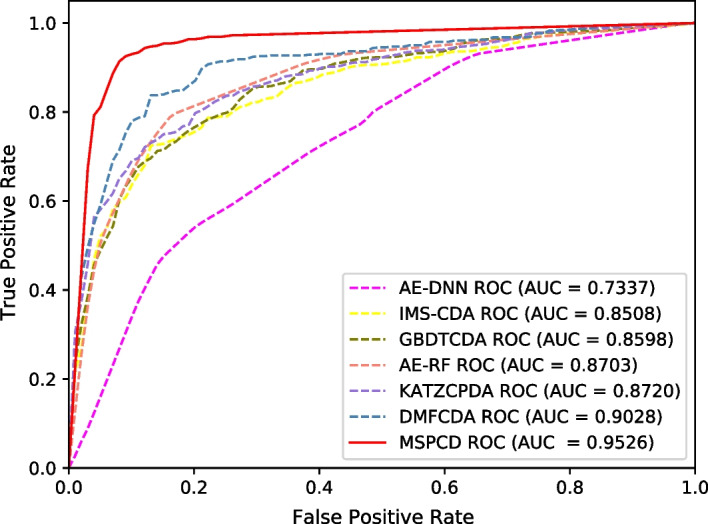
ROC curves performed by seven methods on circR2Disease dataset

In addition, in order to further verify the application capabilities of MSPCD in different datasets, we also compared MSPCD with the above state-of-the-art methods in the circR2Disease database.The ROC curves of these methods are shown in Fig. 5. From the figure, we can see that MSPCD has achieved the best AUC value.The experimental results confirm that MSPCD can be applied to different datasets.

### Analysis effects of the length of high-order feature

In the model, we use neural networks to extract circRNA and disease high-order features, respectively. If the high-order feature’s length is too short, the model will be unable to thoroughly learn the complicated relationship between circRNA and disease. If the high-order feature’s length is too long, the risk of overfitting will be increased. In this section, to study the effect of the length of high-order feature on circRNA and disease associations prediction, we set the length of high-order feature to 8, 16, 32, 64, 128 for experimental comparison.

**Fig. 6:**
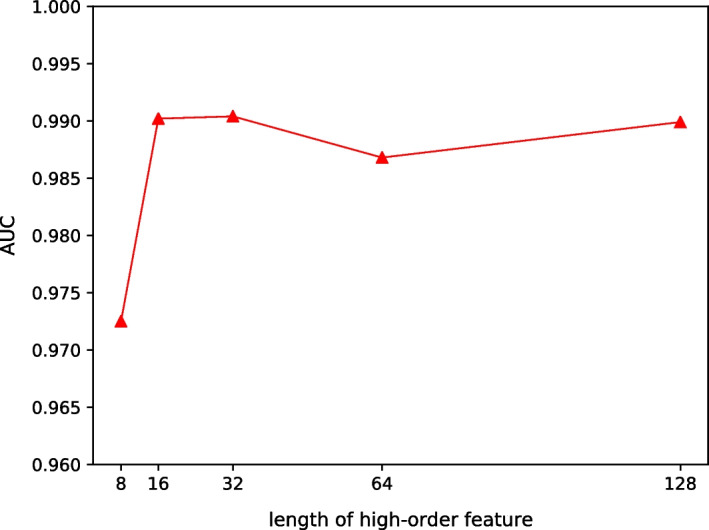
Effects of lengths of high-order feature

The experimental results are shown in Fig. 6. From the figure, we can see that the *AUC* value does not change much when the high-order feature’s length is in the range of 16 to 128. This is because we use regularization to alleviate overfitting.

### Comparison with different classifiers

In predicting circRNA-disease associations, after obtaining circRNA similarity and disease similarity, many previous models directly cascade them to represent each circRNA-disease pair. However, MSPCD firstly uses neural networks to extract high-order features from circRNA similarity and disease similarity. Then, MSPCD employs neural networks to predict circRNA-disease associations. In this section, to verify the effectiveness of hierarchical neural network of our model, we cascade circRNA similarity and disease similarity to represent each circRNA and disease pair, and then directly use several classical classifiers (DNN, RF, SVM) to infer unknown circRNA-disease associations.

**Table 4:** The comparison of different classifiers based on five-fold cross-validation

Classifiers	AUC	Accuracy	Precision	Recall	F1_score
RF	0.8983	0.7828	0.7903	0.7690	0.7794
SVM	0.9697	0.9433	0.9277	0.9617	0.9443
DNN	0.9763	0.9279	0.9326	0.9240	0.9274
MSPCD	0.9904	0.9453	0.9246	0.9702	0.9463

**Fig. 7:**
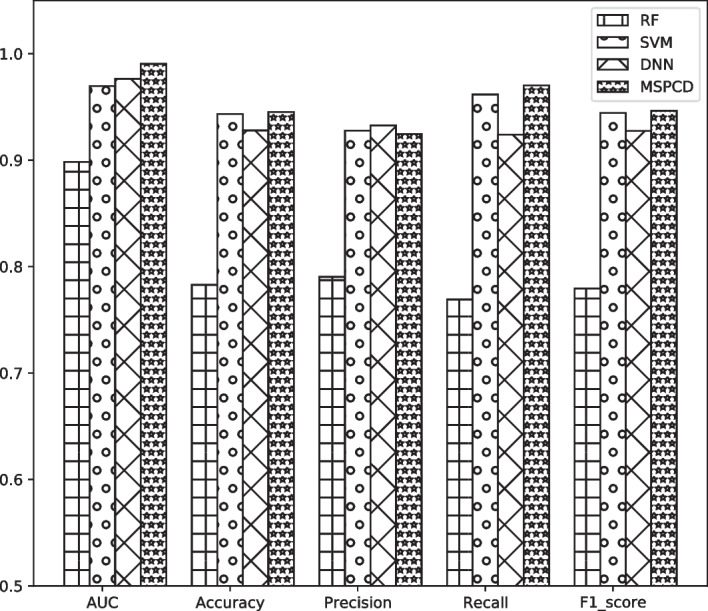
Histograms of the results of different classifiers based on five-fold cross-validation

We have carried out five-fold cross-valid experiments for these classifiers. The experiments are shown in Table 4 and Fig. 7. The *AUC* values of MSPCD, DNN, SVM and RF are 0.9904, 0.9763, 0.9679 and 0.8983 respectively. The experimental results illustrate that hierarchical neural networks used in MSPCD can improve the model’s ability in predicting circRNA-disease association. At the same time, it is worth noting that the *AUC* value of directly using DNN and SVM are also higher than other previous computational methods, which reverifies that it is useful to obtain circRNA similarity and disease similarity by fusing various biological information.

### Case study

To verify the practical capacity of MSPCD in predicting circRNA-disease associations, we perform a case study on the circFunbase dataset. CircFunBase contains 2597 circRNAs ,67 types of diseases and 2984 comfirmed circRNA-disease associations. We used the known circRNA-disease associations in circFunBase to train the MSPCD model, and then predict the remaining 196,944 unknown circRNAs and disease associations. Next, we ranked the unknown circRNAs and disease associations according to the predicted scores. We took out the top 15 circRNAs and disease associations and performed literature validation in the PubMed database. As shown in Table 5, the results show that five of the top 15 circRNA-disease candidate associations are confirmed in PubMed. It’s worth noting that the remaining ones, which have not been confirmed, they are potential circRNA-disease associations to be confirmed.

**Table 5:** Top 15 circRNA-associations predicted by MSPCD on circFunBase dataset

CircRNA	Disease	Evidence (PMID)
hsa_circ_0067997	Gastric cancer	PMID: 30688097
hsa_circ_0082081	Basal cell cancer	Unconfirmed
hsa_circ_0054537	Coronary artery disease	Unconfirmed
hsa_circ_0007534	Cervical cancer	PMID: 31445025
hsa_circ_0004872	Gastric cancer	PMID: 33172486
hsa_circ_0053764	Acute myocardial infarction	Unconfirmed
hsa_circ_0084192	Cervical cancer	Unconfirmed
hsa_circ_0044556	Colorectal cancer	PMID: 32884449
hsa_circ_0028319	Cutaneous squamous cell cancer	Unconfirmed
hsa_circ_0078616	Ovarian aging	Unconfirmed
hsa_circRNA_401801	Colorectal cancer	Unconfirmed
hsa_circ_0007536	Tuberculosis	Unconfirmed
hsa_circ_0030428	Hypertension	Unconfirmed
hsa_circ_0001361	Bladder cancer	PMID: 31705065
hsa_circ_0023546	Cholangiocarcinoma	Unconfirmed

## Conclusion

Understanding the relationship between circRNA and disease will help us recognize the disease mechanism, which is significant for accurate staging and remedy of disease. Additionally, with the formation of many databases about circRNA, it is possible to explore the associations between circRNA and disease by computational methods, which complements for the high cost of biological methods. The previous computational methods have limits because they do not fully consider relevant biological information, resulting in low accuracy of prediction. We developed a method called MSPCD to infer unknown circRNA-disease associations. To obtain similarity of circRNA and disease more accurately MSPCD firstly integrates various biological information. Then, MSPCD extracts high-order features of circRNA and disease by the neural network. Finally, MSPCD utilized DNN to obtain the prediction result. We implemented five-fold cross-validation experiments. the AUC value of MSPCD reached 0.9904 on circFunBase dataset, which outperformed other previous models. The comprehensive experimental results illustrate that MSPCD has a good performance in inferring unknown circRNA-disease associations. In MSPCD, circRNA-disease association prediction is modeled as supervised learning. The sparse supervisory signal leads to limited performance. Self-supervised learning mitigates the effects of sparse supervision signals by pretraining on large-scale dataset without manual annotations. In the future, we will consider applying self-supervised learning to circRNA-disease association prediction.

## Materials and methods

### Problem description

Limited by time and cost, exploring the correlations between circRNAs and human diseases based on biological experiments has encountered many difficulties and bottlenecks. Instead of traditional experiments, computational methods are proved to be an efficient and accurate way to discover the potential connections between circRNAs and diseases.

### Data set

We obtained the circRNA-disease association data from CircFunBase [35]. As a biological information database for circRNAs, CircFunBase provides high-quality functional circRNA resources. We finally extracted 2984 verified circRNA-disease associations, including 2597 circRNAs and 67 diseases, the same as Zheng et al. [36]. Based on this data set, we established the circRNA-disease association matrix $S_d$. If circRNA $c_m$ is related to disease $d_n$, the value of $S_{d}(m, n)$ is 1; otherwise, it is 0.

We also built another circRNA-disease association dataset from circR2Disease [28]. The circR2Disease dataset includes 612 circRNA-disease associations involving 533 circRNAs and 89 diseases. The details of the database are shown in Table 6.

**Table 6:** Statistics of the constructed dataset

Dataset	No. circRNAs	No. diseases	No. known associations	Association density
CircFunBase	2957	67	2984	0.0150
circR2Disease	533	89	612	0.0129

### CircRNA sequence similarity

We acquire the circRNA sequence information from CircFunBase and CircBase (http://www.circbase.org/) and establish the circRNA sequence similarity model based on the Levenshtein distance [37]. Levenshtein distance is a kind of edit distance widely used to measure the similarity between two strings. It is defined as the minimum number of edits to convert a source string into a target string. It only allows three single-character operations: insertion, deletion, and replacement. According to previous relevant researches, the cost of insertion and deletion are set to 1, and the cost of replacement is set to 2. Thus, we can calculate the sequence similarity of circRNA $c_m$ and circRNA $c_n$ using the following formula:

1 $$\begin{aligned} CS\left( c_{m}, c_{n}\right) =\frac{l\left( c_{m}\right) +l\left( c_{n}\right) -cost_{min}}{l\left( c_{m}\right) +l\left( c_{n}\right) } \end{aligned}$$

where $cost_{min}$ indicates the Levenshtein distance between the sequence of $c_m$ and the sequence of $c_n$, and $l(c_m)$ indicates the sequence length of circRNA $c_m$.

### CircRNA functional similarity

Based on the assumption that circRNAs with similar functions are associated with similar diseases, gene ontology (GO) terms, and miRNAs, we also utilize circRNA association information about GO terms and miRNA. These abundant biological materials support us to explore the potential relationship between circRNA and disease entirely. The association data of circRNA-GO and circRNA-miRNA were obtained from CircFunBase through web crawler technology and applied to construct the two’s association matrices, $S_g$ and $S_m$.

The circRNA functional similarity model includes three aspects: disease, Go terms, and miRNA. We use the Jaccard similarity coefficient to measure the similarity score. The disease-based score of circRNA $c_m$ and circRNA $c_n$ can be calculated by the following formula:

2 $$\begin{aligned} CF_{dis}(c_m, c_n)=\frac{|TD(c_m) \cap TD(c_n)|}{|TD(c_m) \cup TD(c_n)|} \end{aligned}$$

where $TD(c_m)$ denotes the binary vector formed by the *m*th row in matrix $S_d$. For $c_m$ and $c_n$, their functional similarity score based on GO terms can be calculated by:

3 $$\begin{aligned} CF_{go}(c_m, c_n)=\frac{|TG(c_m) \cap TG(c_n)|}{|TG(c_m) \cup TG(c_n)|} \end{aligned}$$

where the binary vector $TG(c_m)$ is the *m*th row of the correlation matrix $S_g$. The similarity score of $c_m$ and $c_n$ based on miRNA can be calculated as follows:

4 $$\begin{aligned} CF_{mi}(c_m, c_n)=\frac{|TM(c_m) \cap TM(c_n)|}{|TM(c_m) \cup TM(c_n)|} \end{aligned}$$

where $TM(c_m)$ represents the *m*th row of the association matrix $S_m$. To utilize the above three functional similarities at a comprehensive level, we obtain the final circRNA functional similarity by taking the average value of them, which is computed by:

5 $$\begin{aligned} { CF\left( c_{m}, c_{n}\right) =\frac{CF_{dis}\left( c_{m}, c_{n}\right) +CF_{go}\left( c_{m}, c_{n}\right) +CF_{mi}\left( c_{m}, c_{n}\right) }{3} } \end{aligned}$$

### CircRNA GIP kernel similarity

Gaussian interaction profile (GIP) kernel similarity has been widely applied to extract the network topology information to predict the interaction between biomolecules. According to the biological hypothesis that functionally comparable circRNAs are inclined to be associated with semblable diseases, we calculate the GIP kernel similarity between circRNAs through the circRNA-disease adjacent matrix $S_d$. The calculation formula for circRNA $c_m$ and circRNA $c_n$ is as follows:

6 $$\begin{aligned} CG(c_m, c_n)=\exp \left( -\delta _{c}\Vert TD(c_m)-TD(c_n)\Vert ^{2}\right) \end{aligned}$$

7 $$\begin{aligned} \delta _{c}=\frac{1}{nc} \sum _{m=1}^{nc}\Vert TD(c_m)\Vert ^{2} \end{aligned}$$

where $\delta _c$ is the parameter of kernel bandwidth, and *nc* denotes the number of circRNAs.

### Disease GIP kernel similarity

Many studies have utilized GIP similarity to measure the similarity between diseases because the more similar diseases are, the more similar their correlations with circRNAs. The GIP similarity for disease $d_m$ and disease $d_n$ can be calculated by:

8 $$\begin{aligned} DG(d_m, d_n)=\exp \left( -\delta _{d}\Vert TC(d_m)-TC(d_n)\Vert ^{2}\right) \end{aligned}$$

9 $$\begin{aligned} \delta _{d}=\frac{1}{nd} \sum _{m=1}^{nd}\Vert TC(d_m)\Vert ^{2} \end{aligned}$$

where $\delta _d$ is the width parameter, $TC(d_m)$ denotes the binary vector formed by the *m*th column in the association matrix $S_d$, and *nd* denotes the number of diseases.

### Disease semantic similarity

To construct the semantic similarity model, we use MeSH, a database that supplies a meticulous classification scheme and can be got from (https://www.ncbi.nlm.nih.gov/mesh/). The relationships between diseases can be expressed as a directed acyclic graph (DAG), where nodes represent diseases and edges represent their associations. If a disease *k* is in the DAG of disease *d*, its contribution $G_d(k)$ to *d* is as follows:

10 $$\begin{aligned} G_{d}(k)=\left\{ \begin{array}{ll}\max \left\{ \mu * G_{d}\left( k^{\prime }\right) \mid k^{\prime } \in \text{ children } \text{ of } k\right\} & \text{ if } k \ne d \\ 1 & \text{ otherwise } \end{array}\right. \end{aligned}$$

where $\mu$ is the contribution element. According to the previous paper by Wang et al. [38], we set its value to 0.5. For disease $d_m$ and disease $d_n$, their first semantic similarity model $DS_1(dm, dn)$ can be calculated by:

11 $$\begin{aligned} DS_{1}\left( d_{m}, d_{n}\right) =\frac{\sum _{k \in N_{d_{m}} \cap N_{d_{n}}}\left( G_{d_{m}}(k)+G_{d_{n}}(k)\right) }{\sum _{k \in N_{d_{m}}} G_{d_{m}}(k)+\sum _{k \in N_{d_{n}}} G_{d_{n}}(k)} \end{aligned}$$

where $N_{d_m}$ is defined as the set of diseases in the DAG of disease $d_m$. However, model $DS_1$ only considers the correlation between the layers in disease DAG and ignores the fact that different diseases appear in DAGs at various times. Thus, we construct the second disease semantic model, which calculates the semantic contribution value by the following formula:

12 $$\begin{aligned} G_{d}^{\prime }(k)=\log \left( \frac{n(dis)}{n(DAGs(k))}\right) \end{aligned}$$

where *n*(*DAGs*(*k*)) indicates the number of DAGs including disease *k*, and *n*(*dis*) indicates the total number of all the diseases. We can calculate the semantic similarity score of disease $d_m$ and disease $d_n$ according to the second model as follows:

13 $$\begin{aligned} DS_{2}(d_{m}, d_{n})=\frac{\sum _{k \in N_{d_{m}} \cap N_{d_{n}}}\left( G_{d_{m}}^{\prime }(k)+G_{d{n}}^{\prime }(k)\right) }{\sum _{k \in N_{d_{m}}} G_{d_{m}}(k)+\sum _{k \in N_{d_{n}}} G_{d_{n}}(k)} \end{aligned}$$

### MSPCD model

The MSPCD model employs hierarchical neural networks to reveal the latent assoications between circRNAs and diseases. In this part we will introduce the implementation process of MSPCD in detail.

#### Multi-source information fusion

**Fig. 8:**
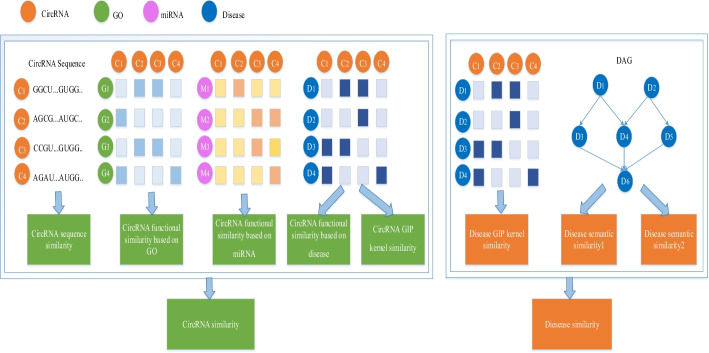
Fuse multi-source data to obtain circRNA similarity and disease similarity

In the aforementioned sections, we have acquired circRNA sequence similarity, circRNA functional similarity, circRNA GIP similarity, disease GIP similarity, and disease semantic similarity. To fully take advantage of data from different sources, we need to fuse the complex similarity information. A better descriptor of the relationship between circRNAs and diseases can help us dig deeper into circRNA-disease associations.The flow chart is showed in Fig. 8.

The integrated similarity of circRNA can be gained by combining circRNA sequence similarity *CS*, circRNA functional similarity *CF*, and circRNA GIP similarity *CG*. In view of the fact that some circRNAs in the circRNA-disease matrix $S_d$ lack the sequence information required for the experiment, we define the binary flag value *FQ* to represent the two opposite situations. If the value of $FQ_{m-n}$ is 1, it means that circRNA $c_m$ and circRNA $c_n$ have sequence similarity. Otherwise, $FQ_{m-n}$ is 0. The fusional similarity matrix of circRNA is defined by the following formula:

14 $$\begin{aligned} \begin{array}{l} CV(c_m, c_n)=\left\{ \begin{array}{ll} \frac{CS(c_m, c_n)+CF(c_m, c_n)}{2} & \text{ if } FQ_{m-n}=1 \\ CG(c_m, c_n) & \text{ if } FQ_{m-n}=0 \end{array}\right. \end{array} \end{aligned}$$

For diseases, we adopt GIP similarity *GS*, semantic similarity $DS_1$ and $DS_2$ to measure the integrated similarity. Since some disease pairs have no matching semantically similar association, the binary flag value *FS* is defined to distinguish different situations. If there is a semantic similarity of disease $d_m$ and $d_n$, the value of $FS_{m-n}$ is 1; otherwise, it is 0. The fusional similarity matrix of disease is defined as follows:

15 $$\begin{aligned} \begin{array}{l} DV(d_m, d_n)=\left\{ \begin{array}{ll} \frac{DS_{1}(d_m, d_n)+DS_{2}(d_m, d_n)}{2} & \text{ if } FS_{m-n}=1 \\ DG(d_m, d_n) & \text{ if } FS_{m-n}=0 \end{array}\right. \end{array} \end{aligned}$$

#### Extract high-order features of circRNA and diseases

There are noise and redundancy in the fundamental features. Designing more efficient features to characterize circRNAs and diseases excellently benefits the performance of the model. Therefore, we take the original characteristics as input and pass them through three layers of fully connected neural networks to extract the dense latent features of circRNAs and diseases, respectively. The activation function rectified linear unit (ReLU) is adopted in the layers mentioned above.

We use *m* to represent the indexes of fully connected layers. The value of *m* in this section is 1, 2, or 3. *e* represents the initial input vectors of circRNAs. $w_{em}$ and $b_{em}$ respectively represent the weight coefficient and bias of the corresponding layer. For circRNAs, the output of each connected layer can be expressed as follows:

16 $$\begin{aligned} O_{em} ={\text {ReLU}}\left( w_{em}O_{e(m-1)}+b_{em}\right) \end{aligned}$$

If *m* is 1, then $O_{e0}$ is equal to e. Likewise, *f* represents the primary input vectors of the diseases. $w_{fm}$ and $b_{fm}$ respectively represent the weight coefficient and bias of the corresponding layer. The output of each connected layer for diseases can be expressed by:

17 $$\begin{aligned} O_{fm} ={\text {ReLU}}\left( w_{fm}O_{f(m-1)}+b_{fm}\right) \end{aligned}$$

$O_{f0}$ is equal to *f* when the value of *m* is 1. The outputs of the third layers, $O_{e3}$ and $O_{f3}$, are the complicated high-level features of circRNAs and diseases.

#### Feature interaction by the dot product

So far, for any circRNA *i* and any disease *j* in the data set, we respectively project their initial feature vectors into N*1-dimensional high-order feature vectors, *CH* and *DH*. The values of *CH* and *DH*’s corresponding positions are multiplied to learn the interaction feature vector *CD* between circRNA *i* and disease *j*, and its dimension is also N*1. We do not directly use interactive characteristics to predict the correlation between circRNA *i* and disease *j* but concatenate them with the high-order feature *CH* of *i* and the high-order feature *DH* of *j*. The generated vector is adopted to represent the circRNA-disease pair $i-j$, which is defined as follows:

18 $$\begin{aligned} VG_{i-j}=\left[ \begin{array}{c} CH_{i} \\ CD_{i-j} \\ DH_{j} \end{array}\right] \end{aligned}$$

**Fig. 9:**
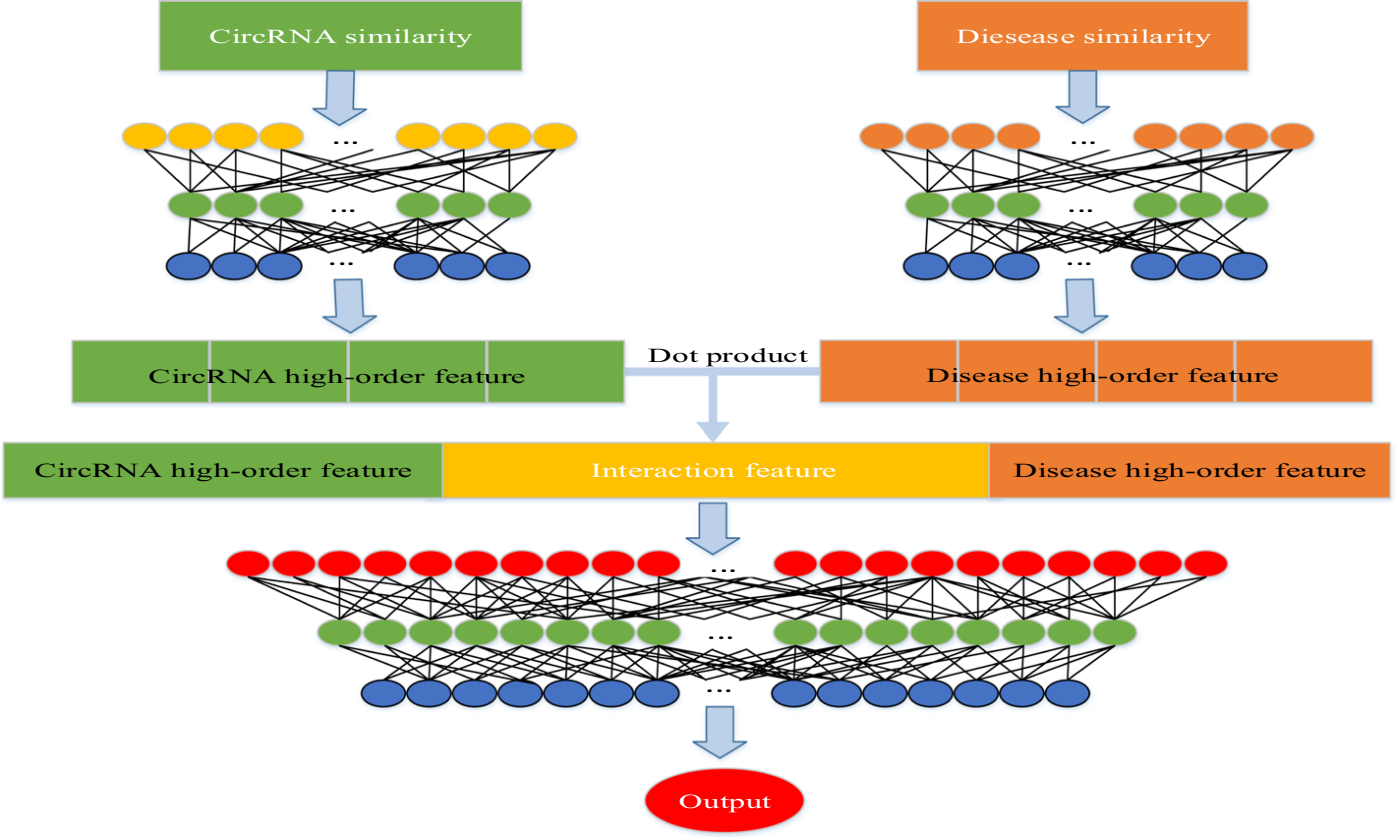
Overview of our proposed MSPCD method for predicting circRNA-disease assoications. Firstly, it takes the similarity of circRNA i and disease j as input and outputs their high-order non-linear features through three fully connected layers. Secondly, we use the dot product to acquire the high-level interactive feature of i and j. The result and the high-order features are concatenated to generate a new vector fed into DNN to finally realize the association prediction between circRNA and disease

#### Predict circRNA-disease associations by DNN

We send the feature vectors acquired above into three fully connected layers. ReLU is utilized as the activation function of the first two fully connected layers, and the last activation function is sigmoid to get the ultimate binary results. *VG* denotes the matrix composed of all the feature vectors generated in the previous step. It is also the input of the fourth fully connected layer. ${\hat{y}}$ denotes the output of the sixth layer, that is, the predicted label values.

19 $$\begin{aligned} O_{4}&= {\text {ReLU}}\left( w_{4}VG+b_{4}\right) \end{aligned}$$

20 $$\begin{aligned} O_{5}&= {\text {ReLU}}\left( w_{5}O_{4}+b_{5}\right) \end{aligned}$$

21 $$\begin{aligned} {\hat{y}}&= {\text {sigmoid}}\left( w_{6}O_{5}+b_{6}\right) \end{aligned}$$

where $w_4$, $w_5$, $w_6$ are the weights of the corresponding connection layer. $b_4$, $b_5$, $b_6$ are the biases of the corresponding connection layer. Before training the model, we notice that the size of the constructed cirRNA-disease association matrix *Sd* is 2597*67, but there are only 2984 associations in *Sd*. To avoid the impact of unbalanced samples, we randomly pick 2984 negative samples from the remaining unverified associations to reach the identical quantity as the positive samples. The selected negative set is not strictly credible, and there may be unproven positive associations. But the influence is negligible because they only occupy a tiny proportion in the whole negative sample set. The flow chart of MSPCD is shown in Fig. 9.

### Evaluation metrics

To evaluate the performance of MSPCD, we choose five-fold cross-validation. First, the dataset is divided into five subsets. Then, four subsets are used for training set and one subset for testing. We repeat that process until all subsets have used for test set. We choose *AUC*, *accuracy*, *precision*, *recall*, and $F1\_score$ as the evaluation indicators and take the average values of five experimental results as the final result. *AUC* is the area under the *ROC* curve and could be regarded as the probability that the predicted score of positive samples is greater than that of negative examples. The remaining indicators are as follows:

22 $$\begin{aligned} Accuracy&= \frac{{\mathrm{{TP + TN}}}}{{TP + FN + FP + TN}} \end{aligned}$$

23 $$\begin{aligned} Precision&= \frac{{\mathrm{{TP}}}}{{TP + FP}} \end{aligned}$$

24 $$\begin{aligned} Recall&= \frac{{\mathrm{{TP}}}}{{TN + FN}} \end{aligned}$$

25 $$\begin{aligned} F1\_score&= \frac{{\mathrm{{2}{\times }{Precision}{\times }{Recall}}}}{{Precision + Recall}} \end{aligned}$$

where *TP* and *TN* are the numbers of circRNA-disease association pairs and non-association pairs which are correctly identified, respectively; *FP* and *FN* are the numbers of circRNA-disease association pairs and non-association pairs which are incorrectly identified, respectively.

We implement MSPCD in Keras 2.2.5. The batch size and learning rate are tuned by grid search in $\left\{ 32, 64,128,256,512\right\}$ and $\left\{ 0.0005,0.001,0.002,0.0025\right\}$, respectively. The dimension of high-order features we search for is $\left\{ 8, 16, 32, 64, 128\right\}$. The number of training epochs is set to 200.

## Data Availability

The data and code used in the current study is available at:https://github.com/dayunliu/MSPCD.

## Abbreviations

ROC: Rceiver operating characteristic
TPR: True positive rate
FPR: False positive rate
AUC: Area under ROC curve
SVM: Support vector machine
RF: Random forest
DAG: Directed acyclic graph
